# Evaluation of costimulatory molecules in dogs with B cell high grade lymphoma

**DOI:** 10.1371/journal.pone.0201222

**Published:** 2018-07-24

**Authors:** Michihito Tagawa, Chihiro Kurashima, Satoshi Takagi, Naoya Maekawa, Satoru Konnai, Genya Shimbo, Kotaro Matsumoto, Hisashi Inokuma, Keiko Kawamoto, Kazuro Miyahara

**Affiliations:** 1 Veterinary Medical Center, Obihiro University of Agriculture and Veterinary Medicine, Obihiro, Hokkaido, Japan; 2 Department of Veterinary Clinical Sciences, Faculty of Veterinary Medicine, Hokkaido University, Veterinary Teaching Hospital, Sapporo, Hokkaido, Japan; 3 Department of Disease Control, Faculty of Veterinary Medicine, Hokkaido University, Sapporo, Hokkaido, Japan; 4 Department of Clinical Veterinary Science, Obihiro University of Agriculture and Veterinary Medicine, Obihiro, Hokkaido, Japan; 5 Research Center for Global Agromedicine, Obihiro University of Agriculture and Veterinary Medicine, Obihiro, Hokkaido, Japan; Cornell University, UNITED STATES

## Abstract

B cell high grade lymphoma is the most common hematopoietic malignancy in dogs. Although the immune checkpoint molecules, programmed death-1 (PD-1) and cytotoxic T-lymphocyte-associated antigen-4 (CTLA-4), and immune checkpoint inhibitors have been evaluated for the treatment of various human lymphoid malignancies, the expression of those molecules and their relationship with prognosis remain unknown in canine lymphoma. The objective of this study was to evaluate the expression of costimulatory molecules on peripheral blood lymphocytes and tumor infiltrating lymphocytes, in addition to associated ligand expression in the lymph nodes of patients with B cell multicentric high grade lymphoma. Eighteen patients diagnosed with B cell high grade lymphoma and nine healthy control dogs were enrolled. Flow cytometric analysis revealed that the expression of PD-1 on CD4+ peripheral and tumor infiltrating lymphocytes and CTLA-4 on CD4+ peripheral lymphocytes was significantly higher in the lymphoma group than in the control group. The expression level of *CD80* mRNA was significantly lower in the lymphoma group than in the control group. In contrast, there were no significant differences in *PD-L1*, *PD-L2*, and *CD86* expression between the groups. Dogs with CTLA-4 levels below the cutoff values, which were determined based on receiver operating characteristic curves, on peripheral CD4+, CD8+, and tumor infiltrating CD4+ lymphocytes had significantly longer survival than dogs with values above the cutoff. Although it is uncertain whether the expression of immune checkpoint molecules affect the biological behavior of canine lymphoma, one possible explanation is that PD-1 and CTLA-4 might be associated with the suppression of antitumor immunity in dogs with B cell high grade lymphoma, particularly through CD4+ T cells.

## Introduction

Lymphoma is one of the most frequently occurring malignant neoplasms in dogs, and accounts for approximately 7–24% of all canine neoplasms and 83% of all hematopoietic malignancies [1]. The multicentric form of B cell lymphoma is most common, with a high percentage of cases involving the lymphoreticular system, which includes the lymph nodes, liver, spleen, and bone marrow [2]. Various combination chemotherapies have been reported to induce remission in approximately 80–95% of dogs; however, the majority of dogs will relapse within one year of starting treatment and overall median survival times are limited to 10–12 months [1, 3]. Specifically, diffuse large B cell lymphoma (DLBCL) is the most common subtype of the canine multicentric form of B cell lymphoma, and its relevance as a spontaneous model for human DLBCL has been confirmed by molecular and morphological approaches [4].

T cell functions are regulated by several immune checkpoint molecules [5]. Programmed cell death 1 (PD-1) is an immune checkpoint molecule that is expressed on both activated and exhausted T cells. PD-1 has two ligands, namely PD-L1 (B7-H1) and PD-L2 (B7-DC). PD-L1 is widely expressed on non-hematopoietic cells including tumor cells and antigen-presenting cells, whereas PD-L2 expression is restricted to B cells, macrophages, and dendritic cells [6]. The interactions between PD-1 and PD-Ls provide a negative stimulus for antigen-induced T cell activation [7]. Cytotoxic T-lymphocyte antigen-4 (CTLA-4), which is expressed on the surface of activated T lymphocytes, is another immune checkpoint molecule that transmits signals to inhibit T cell activation, through binding to the its ligands, CD80/86, which are expressed on antigen-presenting cells [8]. These immune checkpoint molecules including PD-1 and CTLA-4 are highly expressed on tumor infiltrating and peripheral lymphocytes, and their ligands are up-regulated in many human cancers [9, 10]. Immune checkpoint molecules are believed to represent an important mechanism through which tumor cells evade the host immune system, and evidence of immune dysregulation has been reported in several human cancers [9, 10, 11]. Several reports have demonstrated that the expression of these molecules is significantly correlated with a worse prognosis [10, 11]. In addition, immune checkpoint inhibitors such as anti-PD-1, anti-PD-L1, and anti-CTLA-4 antibodies have shown promising effects for several human malignancies [12]. In veterinary medicine, previous studies have revealed that these immune checkpoint molecules including PD-1 and CTLA-4 are also highly expressed, with PD-L1 being up-regulated, in several cancers [13, 14, 15, 16].

Recently, several reports have examined the expression of immune checkpoint molecules on peripheral blood and/or tumor-infiltrating T cells in hematological malignancies, and its correlation with prognosis has been discussed [17, 18, 19]. In addition, those inhibitors have also been evaluated for the treatment of various lymphoid malignancies [17]. PD-1 is expressed by tumor infiltrating lymphocytes in several types of lymphoma [18] and PD-Ls are also expressed on lymphoid tumor cells and nonmalignant tumor infiltrating cells, primarily macrophages [19, 20]. In addition, it was demonstrated that PD-L1 expression on tumor cells is correlated with a poor prognosis in B cell malignancies [19]. Several clinical trials of checkpoint inhibitors have been conducted, and these have shown remarkable efficacy for some refractory hematologic malignancies [17]. Recently, canine multicentric high grade lymphoma has been shown to harbor many similarities to human non-Hodgkin’s lymphoma, and thus it is considered to be an attractive model for human studies [4, 21]. Therefore, these immune checkpoint molecules might represent new therapeutic targets for the treatment of canine lymphoma. However, there are only a few reports regarding immune checkpoint molecules in dogs with lymphoma, and their association with the disease is relatively unknown [13, 14]. The aim of this study was to evaluate the expression of costimulatory molecules on peripheral blood lymphocytes and tumor infiltrating lymphocytes, as well as the expression of their ligands on tumor cells, from patients with B cell multicentric high grade lymphoma to assess immune status in these dogs.

## Materials and methods

### Study population

Eighteen dogs with B cell multicentric high grade lymphoma (lymphoma group) and nine clinically healthy controls (control group) were enrolled in this prospective study. For patients in the lymphoma group, lymphoma was cytologically or histologically confirmed in a veterinary medical center at Obihiro University of Agriculture and Veterinary Medicine between October 2015 and August 2017. Staging of lymphoma was performed according to the WHO staging system [22] including thoracic radiography based on three views, abdominal ultrasound, and hematological examinations. Substage ‘a’ was defined as no clinical signs and substage ‘b’ was defined as systemic signs related to lymphoma including mild-to-moderate severity of gastrointestinal disorders or weight loss. The immunophenotype was determined by PCR antigen receptor rearrangement using fine needle aspirate (FNA) samples. All dogs received no prior chemotherapy treatment except for one relapsed case. Dogs in the control group were age-matched healthy patients who came to the hospital for medical examinations. This study was approved by the Institutional Animal Care and Use Committees at the Obihiro University of Agriculture and Veterinary Medicine (Permission number: 27–143, 28–7, and 29–3).

### Cell preparation

Heparinized peripheral blood was obtained from all patients and healthy controls. All heparinized blood samples were diluted with an equal volume of 0.9% saline, and peripheral blood mononuclear cells (PBMCs) were separated by Ficoll-Paque (Lymphoprep; Axis-Shield PoC AS, Oslo, Norway) density gradient centrifugation [23]. Following centrifugation at 800 × g for 20 min at room temperature, PBMCs were collected and washed twice with 0.9% saline. FNA samples were taken from palpable lymph nodes, and lymph nodes cells (LNCs) were suspended in 500 μl of saline. LNCs were centrifuged for 15 min at 1,500 rpm, and the supernatant was discarded. Cells were resuspended in 1 ml of VersaLyse (Beckman Coulter, Marseille Cedex, France) and incubated for 5 min at room temperature in the dark. Following centrifugation, LNCs were washed and resuspended in 0.9% saline.

### Flow cytometric analysis

For flow cytometric analysis, cells were pelleted by centrifugation for 5 min at 1,500 rpm and washed with phosphate-buffered saline (PBS) containing 10% normal goat serum. Between 0.5 × 10^6^ and 1 × 10^6^ cells were stained using CD4, CD8, PD-1, and CTLA-4 antibodies for 30 min at 37 °C, as previously described [23]. The following antibody conjugates were used: rat anti-canine CD4 monoclonal antibody (mAb): YKIX302.9 prelabeled with fluorescein isothiocyanate; rat anti-canine CD8 mAb: YCATE55.9 prelabeled with r-phycoerythrin (dilution rate 100:2.5, AbD Serotec, Raleigh, NC, USA); mouse anti-human CTLA-4 mAb: ANC152.2 (final concentration: 0.001 mg/ml, Ancell Corporation, Bayport, MN, USA) [16]; goat anti-human PD-1 polyclonal antibody (pAb): BAF1086 (0.005 mg/ml, R&D Systems, Minneapolis, MN, USA) [15, 24, 25]. Additionally, dye/isotype-matched antibodies were included in all experiments as controls. The cells were immediately analyzed (percentage and mean fluorescence intensity [MFI]) using BD FACS Cant and FACS Diva software (BD Biosciences, San Jose, CA, USA). Lymphocytes were first gated through forward and side scatter and then PD-1 and CTLA-4 expression was analyzed in CD4+ and CD8+ lymphocytes populations. A minimum of 10,000 cells were analyzed for each sample. Detection limits were set based on the isotype controls such that less than 1% of cells were positive. (S1 Fig).

### RNA extraction and cDNA synthesis

Total RNA was isolated from LNCs using TRIzol^®^ Reagent (Thermo Fisher Scientific Inc., Waltham, MA, USA) according to the manufacturer’s protocol. After the isolation of total RNA, genomic DNA contamination was removed from the isolated RNA using recombinant DNase I (TURBO DNA-free^™^ Kit; Thermo Fisher Scientific Inc.) according to the manufacturer’s recommended procedure. The RNA concentration was measured using a NanoDrop Lite (Thermo Fisher Scientific Inc.), and the purity of the extracted RNA was in the range of 1.8–2.0, as determined by absorbance ratios of A_260_/A_280_. The purified RNA was eluted in nuclease-free water and stored at −80 °C until further use.

cDNA was synthesized from 500 ng of total RNA using PrimeScript^™^ RT Master Mix containing Oligo dT primers and random primers (Takara Bio Inc., Kusatsu, Japan) according to the manufacturer’s protocol.

### Quantification of *PD-L1*, *PD-L2*, *CD80*, and *CD86* mRNA by real-time RT-PCR

RT-PCR amplification was performed to measure the levels of *PD-L1*, *PD-L2*, *CD80*, and *CD86* mRNA. Primers used for quantitative PCR were designed using Primer3Plus (http://www.bioinformatics.nl/cgi-bin/primer3plus/primer3plus.cgi/) based on canine GenBank sequences or previous reports (Table 1). All primer sets were designed to specifically amplify a region encompassing two exons of each gene. The NormFinder algorithm (https://www.moma.dk/normfinder-software) was used to identify the most stable gene among three candidate reference genes (*ACTB*, *RPL13A*, and *RPL32*) [26], and RPL13A was chosen as the reference gene. PCR products were subjected to Hokkaido System Science (Sapporo, Japan), and the specificities of these primers were confirmed by sequencing analysis. Nucleotide sequence results were confirmed using the BLAST search program (http://www.ncbi.nlm.nih.gov/blast/Blast.cgi) for comparison of other known sequences. Each real-time RT-PCR reaction was performed in a 20-μl total volume, containing 200 nM of each primer, 1 μl of cDNA, and GoTaq qPCR Master Mix (Promega Corporation, Madison, WI, USA), using a StepOne Real-Time PCR System (Applied Biosystems, South San Francisco, CA, USA). Initial incubation at 95 °C for 2 min was followed by 40 cycles consisting of denaturation at 95 °C for 3 sec and annealing/extension at 60 °C for 30 sec. A melt curve (60–95 °C) was generated at the end of each run to verify specificity. Amplification efficiency for each reaction was tested by the series dilution method. Absolute quantification of each mRNA was performed by converting sample cycle threshold values to a concentration (copies/μl), based on standard curves, which were generated using 10-fold serial dilutions (10^7^–10^3^ copies/μl) of each gene amplicon. The target amount was then divided by *RPL13A* levels to obtain a normalized target value. All samples were evaluated in triplicate.

**Table 1:** Primer sequences for canine *PD-L1*, *PD-L2*, *CD80*, *CD86*, *ACTB*, *RPL13A*, and *RPL32* genes.

Target gene	Primer name	Sequence (5'–3')	Product length (bp)	GenBank accession No.
**PD-L1**	f600	TGGCAAAACCACCATCACTA	214	NM001291972
	r813	CAGGAAAGGTCCCAGAATCA		
**PD-L2**	F2	AAGACCTCCCAAGGCCTCTA	172	XM847012
	R2	CATGAAGCAGCCAGTTTGAA		
**CD80**	F	ATGGATTACACAGCGAAGTGGAGAA	323	AF106824
	R	AGGCGCAGAGCCATAATCACGAT		
**CD86**	F	ATGTATCTCAGATGCACTATGGAAC	221	AF106824
	R	TTCTCTTTGCCTCTGTATAGCTCGT		
**ACTB**	F	CCGCGAGAAGATGACCCAGA	237	Z70044
	R	GTGAGGATCTTCATGAGGTAGTCGG		
**RPL13A**	F	GCCGGAAGGTTGTAGTCGT	87	AJ388525
	R	GGAGGAAGGCCAGGTAATTC		
**RPL32**	F	TGGTTACAGGAGCAACAAGAA	100	XM848016
	R	GCACATCAGCAGCACTTCA		

### Statistical analysis

Sexes within each group were compared using the chi-squared test. Continuous variables such as age and PD-1 and CTLA-4 parameters were analyzed by performing Mann-Whitney U tests. Survival time was measured as the interval between the sampling day, usually the start of treatment, and death due to lymphoma. Dogs alive at the end of the study were censored for survival analysis. The survival curves were examined by the Kaplan-Meier method, which were compared using the log-rank test. Receiver operating characteristic (ROC) curve analysis was used to determine the optimal cutoff values of continuous variables used for the prediction of a survival time exceeding a median value. A minimum area under the curve (AUC) of 0.7 was required for the ROC model to be considered. *X*^2^ Fisher’s exact test was used to evaluate categorical values. All analyses were performed using JMP 13 (SAS Institute Inc., Cary, NC, USA). Differences were considered statistically significant if the P value was less than 0.05.

## Results

### Characteristics of the study population

In the lymphoma group, the median age at sampling was 9.5 years (range, 3–14 years). Eleven dogs were male (five were castrated) and seven dogs were female (five were spayed). In the control group, the median age at sampling was 8.5 years (range, 1–12 years). Three dogs were male (one was castrated) and six dogs were female. There were no significant differences in terms of age or sex between the two groups. According to WHO staging, one dog was classified as stage II, five as III, six as IV, and six as V. Eleven dogs were classified as substage a and seven as b. Only two dogs were treated with prednisolone before sampling. Ten dogs were treated with a CHOP-based protocol and five dogs with prednisolone alone. The others were treated with L-asparaginase (as outlined in Table 2). Euthanasia was performed only for one dog, which was in extremis, and all other cases died naturally. The overall median survival time was 121 days (range, 1–340 days). Two dogs were still alive at the end of the study, representing survival of 191 and 208 days.

**Table 2:** Clinical parameters of 18 dogs diagnosed with B cell lymphoma.

Clinical parameters	Case	Percentage (%)
**Age**		
< 10	9	50
≥ 10	9	50
**Sex**		
Male	11	61.1
Female	7	38.9
**Stage**		
II–III	6	33.3
IV–V	12	66.7
**Substage**		
a	11	61.1
b	7	38.9
**Therapy**		
CHOP-based	10	55.6
L-asparaginase	3	16.7
Prednisolone	5	27.8

### PD-1 and CTLA-4 expression on T cells from PBMCs and LNCs

**Fig 1:**
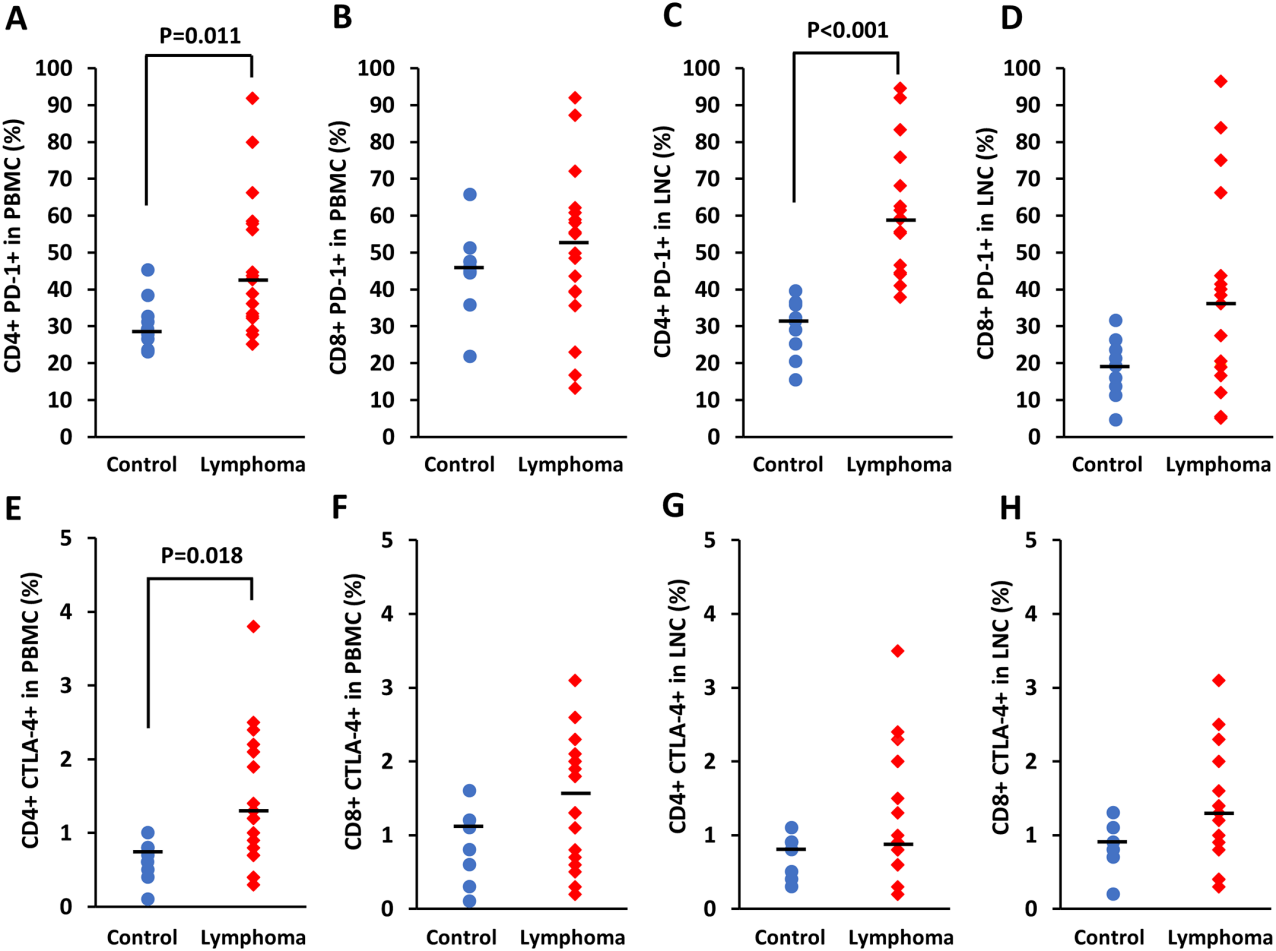
Proportion of PD-1+ and CTLA-4+ cells in CD4+ and CD8+ lymphocyte populations from dogs with B cell lymphoma and control animals. Results were obtained by performing flow cytometry. Each dot represents a patient and the bar represents the median. P values are shown.

PBMCs were available from all dogs, and LNCs were available from 17 of 18 dogs with lymphoma and from all nine control dogs. The proportion of PD-1- and CTLA-4-expressing cells was calculated as the percentage of PD-1+ or CTLA-4+ cells in CD4+ and CD8+ lymphocyte subtypes for each group. The proportions of PD-1+ cells in CD4+ lymphocyte populations obtained from PBMCs and LNCs were significantly higher in the lymphoma group (PBMCs, mean ± standard deviation: 46.67% ± 18.52%; LNCs, 61.29% ± 16.99%) than in the control group (PBMCs, 30.79% ± 7.18%, P = 0.011, Fig 1A; LNCs, 29.48% ± 7.90%, P < 0.001, Fig 1C). Regarding, PD-1 expression in CD8+ lymphocytes obtained from PBMCs and LNCs, there were no significant differences between the groups (Fig 1B and 1D). The proportion of CTLA-4+ cells in CD4+ lymphocyte populations obtained from PBMCs was significantly higher in the lymphoma group (1.40% ± 0.92%) than in the control group (0.62% ± 0.26%, P = 0.018, Fig 1E). Regarding CTLA-4 expression in CD8+ lymphocytes obtained from PBMCs and CD4+ and CD8+ lymphocytes obtained from LNCs, there were no significant differences between the groups (Fig 1F, 1G and 1H). MFIs of PD-1 on the surface of CD4+ lymphocytes obtained from PBMCs and LNCs were significantly higher in the lymphoma group (PBMCs, 1169 ± 573, P = 0.004; LNCs, 2655 ± 1116, P < 0.001) than in the control group (PBMCs, 617 ± 262; LNCs, 933 ± 375; S2 Fig).

### *PD-L1*, *PD-L2*, *CD80*, and *CD86* mRNA expression levels in LNCs

**Fig 2:**
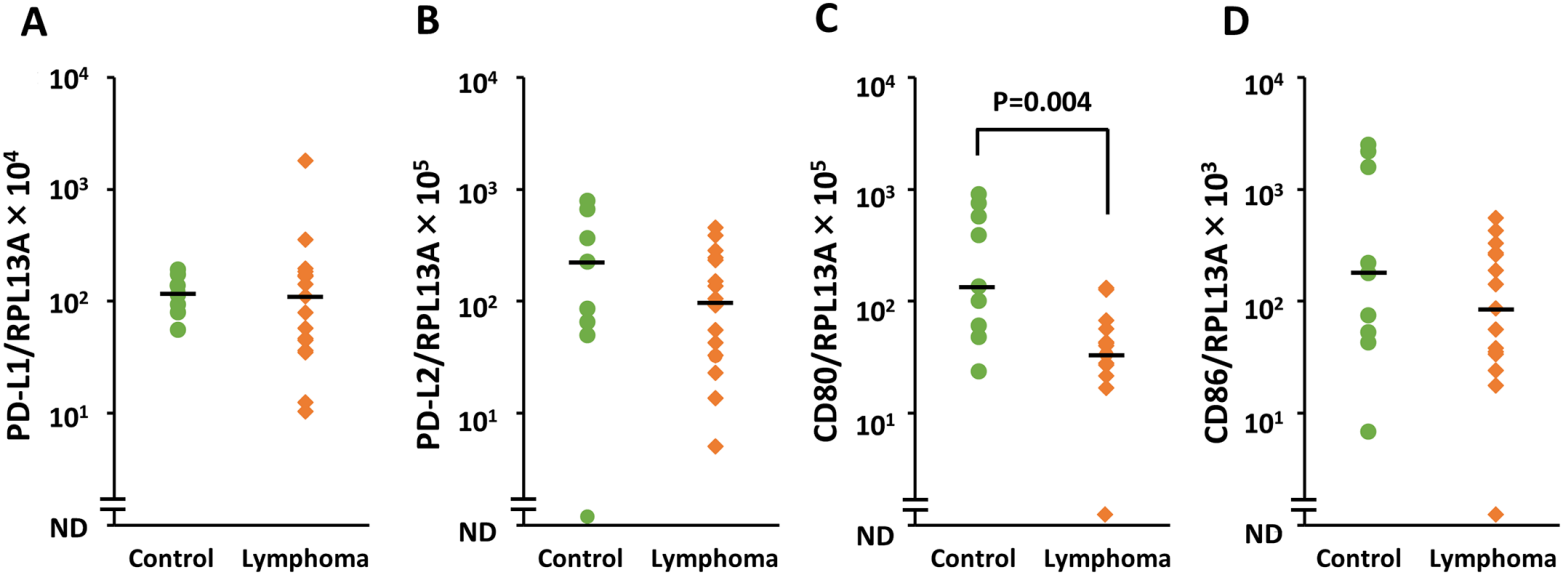
Relative quantity of *PD-L1*, *PD-L2*, *CD80*, and *CD86* transcript levels in lymph node cells from dogs with B cell lymphoma and control animals. Expression of each target mRNA was obtained by real-time PCR, normalizing to the target gene *RPL13A*. Each dot represents a patient and the bar represents the median. P value is shown. ND, not detected.

Based on sequence analysis, PCR products of *PD-L1*, *PD-L2*, *CD80*, *CD86*, *ACTB*, *RPL13A*, and *RPL32* were shown to have 100% identity with each reference gene. By analyzing the linearity of amplification for successively diluted cDNA, the efficiency for all the reactions was determined. The slopes ranged from −3.37 to −3.84, the *R*^2^ values ranged from 0.96 to 0.99, and the efficiencies ranged from 82.1% to 98.0%. The expression levels of *PD-L1*, *PD-L2*, *CD80*, and *CD86* mRNA in LNCs were evaluated by real-time PCR. RNA samples were available from 17 of 18 dogs with lymphoma and for all nine control dogs. The *PD-L1* transcript was expressed in all dogs, whereas *PD-L2*, *CD80*, and *CD86* were detected in 25 of 26 dogs. The expression level of *CD80* was significantly lower in the lymphoma group than in the control group (P = 0.004, Fig 2C). In contrast, there were no significant differences in terms of *PD-L1*, *PD-L2*, and *CD86* expression between the groups (Fig 2A, 2B and 2D).

### Relationship between expression of immune checkpoints and survival time

**Fig 3:**
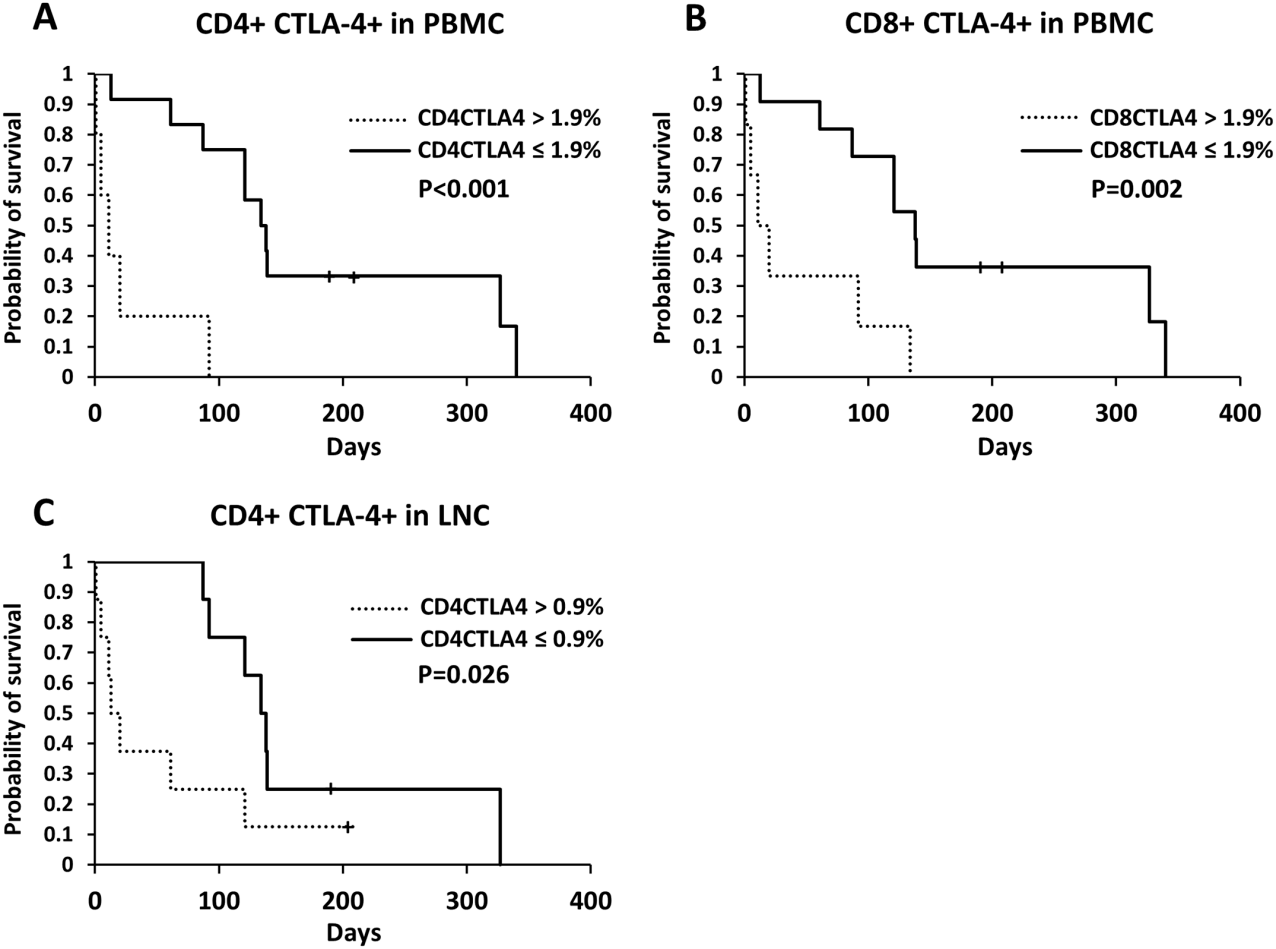
Kaplan-Meier curves of survival time in dogs with B cell lymphoma according to each cutoff value based on CTLA-4 expression. Expression of each marker was determined in CD4+ and CD8+ cell populations. Cutoff and P values are shown. + censored case.

Optimal cutoff values were determined based on each ROC curve (Table 3). Regarding CTLA-4 expression on CD4+ and CD8+ lymphocytes, dogs with levels below the cutoff value had significantly longer survival time than the dogs with values above the cutoff (CD4+ CTLA-4+ in PBMCs, median survival times below and above the cutoff = 138 days and 11 days, respectively, P < 0.001, Fig 3A; CD8+ CTLA-4+ in PBMCs, 138.5 days and 15.5 days, respectively, P = 0.002, Fig 3B; CD4+ CTLA-4+ in LNCs, 138 days and 16.5 days, respectively, P = 0.026, Fig 3C). In addition, for CTLA-4+ cells in CD8+ populations and PD-1+ cells in CD4+ lymphocytes obtained from LNCs, dogs with levels below the cutoff value had significantly longer survival time than dogs with values above the cutoff (CD8+ CTLA-4+ in LNCs, 121 days and 44 days, respectively, P = 0.048; CD4+ PD-1+ in LNCs, 127.5 days and 13 days, respectively, P = 0.023); however, their AUC values were below 0.7 (data not shown). Except for CD4+ PD-1+ in LNCs, the expressions of PD-1+ on lymphocytes did not correlate with survival time. Regarding mRNA expression, no correlation was found with survival time (data not shown). The clinical features and treatment modality for dogs with each parameter level below or above the cutoff values are summarized in S1 Table. Higher proportions of CD4+ PD-1+ (P = 0.047), CD4+ CTLA-4+ (P = 0.003), and CD8+ CTLA-4+ (P = 0.013) cells in PBMCs were significantly correlated with substage b.

**Table 3:** Cutoff and area under the curve (AUC) values obtained from receiver operating characteristic (ROC) curves for each parameter.

Parameters	Cutoff	AUC	95% CI	P value
**PBMCs**				
CD4+ PD-1+	56.2	0.575	0.261–0.889	0.419
CD8+ PD-1+	55.2	0.638	0.353–0.922	0.400
CD4+ CTLA-4+	1.9	0.781	0.538–1.000	0.011
CD8+ CTLA-4+	1.9	0.806	0.572–1.000	0.021
**LNCs**				
CD4+ PD-1+	75.9	0.639	0.345–0.932	0.183
CD8+ PD-1+	38.5	0.861	0.672–1.000	0.003
CD4+ CTLA-4+	0.9	0.785	0.543–1.000	0.043
CD8+ CTLA-4+	2.3	0.535	0.235–0.834	0.559
**mRNA**				
PD-L1	182.7	0.500	0.179–0.821	0.228
PD-L2	232.4	0.458	0.133–0.783	0.562
CD80	67.7	0.472	0.164–0.781	0.390
CD86	142.5	0.472	0.161–0.784	0.711

PBMC, peripheral blood mononuclear cells; LNCs, lymph nodes cells.

## Discussion

In this study, we evaluated the expression of immune checkpoint molecules including PD-1 and CTLA-4 and their ligands in canine lymphoma patients. In these lymphoma samples, significantly higher expression of CTLA-4 was observed on CD4+ lymphocytes obtained from PBMCs. In addition, the expression of PD-1 on CD4+ lymphocytes obtained from PBMCs and LNCs was also significantly higher in the lymphoma group. CTLA-4, a costimulatory receptor expressed on the surface of activated T cells, is a negative regulator of T cell activation. PD-1 is also an immunoinhibitory receptor expressed by chronically stimulated CD4+ and CD8+ T cells after T cell activation [8, 9]. Up-regulation of the expression of checkpoint regulators plays an important role in the evasion of immune surveillance by malignant cells [17]. In humans, PD-1 expression has been evaluated in different lymphoma types including B cell lymphoma [27, 28, 29, 30]. CTLA-4 is highly expressed on tumor-infiltrating lymphocytes in lymphoid malignancies [31, 32]. There have also been some reports on the PD-1/PD-L1 axis in dogs with different malignancies [13, 14, 33]. Recently, high expression of PD-1 on tumor infiltrating T cells was reported in dogs with B and T cell lymphoma [34]. In this study, significantly higher expression of PD-1 was observed on CD4+ T cells obtained from LNCs from dogs with lymphoma, and CD8+ T cells tended to up-regulate these molecules. In addition, PD-1 expression on peripheral CD4+ T cells obtained from dogs with lymphoma was significantly higher compared to that in control animals. In veterinary medicine, although previous studies revealed high expression of CTLA-4 in dogs with oligodendroglioma and histiocytic sarcoma [15, 16], the expression of this marker in lymphoma had not previously been reported. We found that CTLA-4 expression on CD4+ T cells obtained from PBMCs was significantly higher in the lymphoma group than in the control group. Lymphocytes including T and B cells are the major cells of the adaptive immune system. CD8+ T cells destroy virus-infected cells and tumor cells. CD4+ T lymphocytes have no cytotoxic or phagocytic activity and cannot kill infected cells or clear pathogens; however, they do manage the immune response by directing other cells to perform these tasks [35]. This study suggested that PD-1 and CTLA-4 might be associated with the suppression of antitumor immunity in dogs with B cell lymphoma, and particularly through CD4+ T cells.

To further evaluate the immune checkpoint pathway, mRNA expression levels of *PD-L1*, *PD-L2*, *CD80*, and *CD86* were measured in LNCs. Although there was no significant difference, *PD-L1* expression was variable between the lymphoma group and the control group. Maekawa et al. observed that canine DLBCL samples did not express PD-L1 [13]. Shosu and Kumar et al. reported the expression of PD-L1 in few lymphoma tissues and cell lines [14, 33]. Our results are in agreement with those from the previous reports. In humans, the proportion of DLBCL tumor cells that express PD-L1 was ranged from 10.0 to 49.0% [36, 37]. Further studies are needed to clarify the expression of PD-L1 and the role of the PD-1/PD-L1 axis in canine B cell lymphoma using a large sample size. Interestingly, low expression of *CD80* was observed in canine lymphoma samples. CD80, also known as B7.1, is a coregulatory receptor expressed on the surface of antigen presenting cells, activated T cells, and myeloid-derived suppressor cells. Full activation of T cells requires binding of the CD28 receptor by CD80 or CD86 on antigen-presenting cells [38]. It was reported that CD80 is expressed in the majority of human DLBCL cases and is also present on nonmalignant stromal cells [39]. In contrast, one study revealed that acute lymphoblastic leukemia cells have low expression of costimulatory molecules including CD80 and suggested that this probably contributes to the absence of a host T cell-stimulated immune response [40]. This is the first report of the evaluation of *CD80* expression in canine B cell lymphomas. Further understanding of the expression of this marker, as well as other immune coregulatory molecules that control immune cell function, will be needed to better understand the complexity and functional implications of the tumor microenvironment in canine B cell high grade lymphoma.

In this study, CTLA-4 expression on peripheral CD4+ and CD8+ lymphocytes and CD4+ lymphocytes obtained from LNCs was found to be associated with poor prognosis in canine B cell high grade lymphomas. Especially, high expression of CTLA-4 in PBMCs was significantly correlated with substage b, which has been identified as an indicator of poor prognosis. Although the prognostic role and clinical application of CTLA-4 in tumors are still controversial, increased CTLA-4 expression on T cells contributes to poor prognosis in various cancers including DLBCL [41, 42]. In addition, CTLA-4 is a negative immunomodulatory factor that is known to be expressed by effector regulatory T cells, which mainly suppress antitumor immunity, but not by non-Tregs [41]. It was thought that expression of CTLA-4 on T cells might be associated with a worse prognosis and might represent a prognostic factor for canine B cell high grade lymphoma. In contrast, PD-1 expression on peripheral and tumor infiltrating T cells was not associated with prognosis. In humans, the relationship between PD-1 expression and prognosis in lymphoid malignancies is also complex [43]. To the best of our knowledge, this is the first report to explore the relationship between immune checkpoint molecules and B cell lymphoma prognosis in dogs. However, we did not control for treatment and stage/substage for each analysis, and the small sample size of this study might affects the reliability of the results. Further studies using larger cohorts and controlled patients are needed to evaluate the association between immune checkpoint molecule expression and prognosis in canine B cell high grade lymphomas.

PD-1-and CTLA-4-blocking antibodies (ipilimumab and nivolumab) were shown to produce robust responses and improve overall survival in human patients with various lymphoid malignancies [43]. Interestingly, in canine visceral leishmaniasis, high level of PD-1 on T cells and *in vitro* efficacy of monoclonal antibodies that block PD-1 and its ligands have been reported [24, 25]. Recently, in veterinary medicine, a pilot study demonstrated promising anticancer activity with tolerable toxicity profiles using an anti-PD-L1 antibody for canine malignancies [44]. The results of the present study indicated that such immunotherapy regimens might also be effective for patients with canine B cell high grade lymphoma.

Several limitations should be noted when interpreting the results of this study. First, the small sample size for each analysis might limit the power to detect differences between groups. Therefore, our findings should be verified in a larger population, as this study represents a pilot study. Second, the treatment modality for the dogs with lymphoma was not unified. A future prospective study using standardized treatment is thus necessary for adequate evaluation. Another limitation of our method is the lack of protein quantification of the immune checkpoint ligands. Finally, it remains unclear whether the expression of immune checkpoint molecules on tumor cells or other nonmalignant cells is associated with the anti-tumor immune response and clinical outcome in canine lymphoma patients. Future functional analyses are necessary to elucidate the complex roles of this pathway.

In summary, this study indicates that PD-1 expression is up-regulated on both peripheral and tumor infiltrating T cells and that CTLA-4 expression is up-regulated on CD4+ T cells from peripheral blood obtained from dogs with B cell high grade lymphoma. CTLA-4 expression on T cells was also associated with a poor prognosis. Our findings support the hypothesis that these molecules might represent new therapeutic targets for the treatment of canine B cell high grade lymphoma, and could serve as prognostic factors. Future investigations and clinical trials are needed to develop therapeutic strategies based on the mechanisms of immune checkpoint molecule-induced tumor immune evasion and the modulation of host immune responses for canine B cell high grade lymphoma.

## Supporting information

S1 Fig Analysis of a PBMC sample for PD-1 expression in the CD4+ and CD8+ lymphocyte gate. A representative sample of forward versus side scatter identified the predominant lymphocyte population captured in region P1. The proportions of CD4 (P2) and CD8 (P3) cells in P1 are indicated (A). The proportions of PD-1 expression cells in P2 and P3 are indicated in the middle panels (B). The under panels show each isotype control (C). SSC, side scatter; FSC, forward scatter; PE, phycoerythrin; FITC, fluorescein isothiocyanate; APC, allophycocyanin.
(TIF)

S2 Fig MFI of PD-1+ and CTLA-4+ cells in CD4+ and CD8+ lymphocyte populations from dogs with B cell lymphoma and control animals. Each dot represents a patient and the bar represents the median. P values are shown.
(TIF)

S1 Table Clinical features and treatment modality comparisons between dogs with each parameter level below and above the cutoff values. Chemotherapy included CHOP-based and L-asparaginase.
(XLSX)
