# Supplementary figures and images for: Evaluation of costimulatory molecules in dogs with B cell high grade lymphoma
Source: PLoS One. 2018 Jul 24;13(7):e0201222. doi: 10.1371/journal.pone.0201222 (PMC6057677; doi:10.1371/journal.pone.0201222)

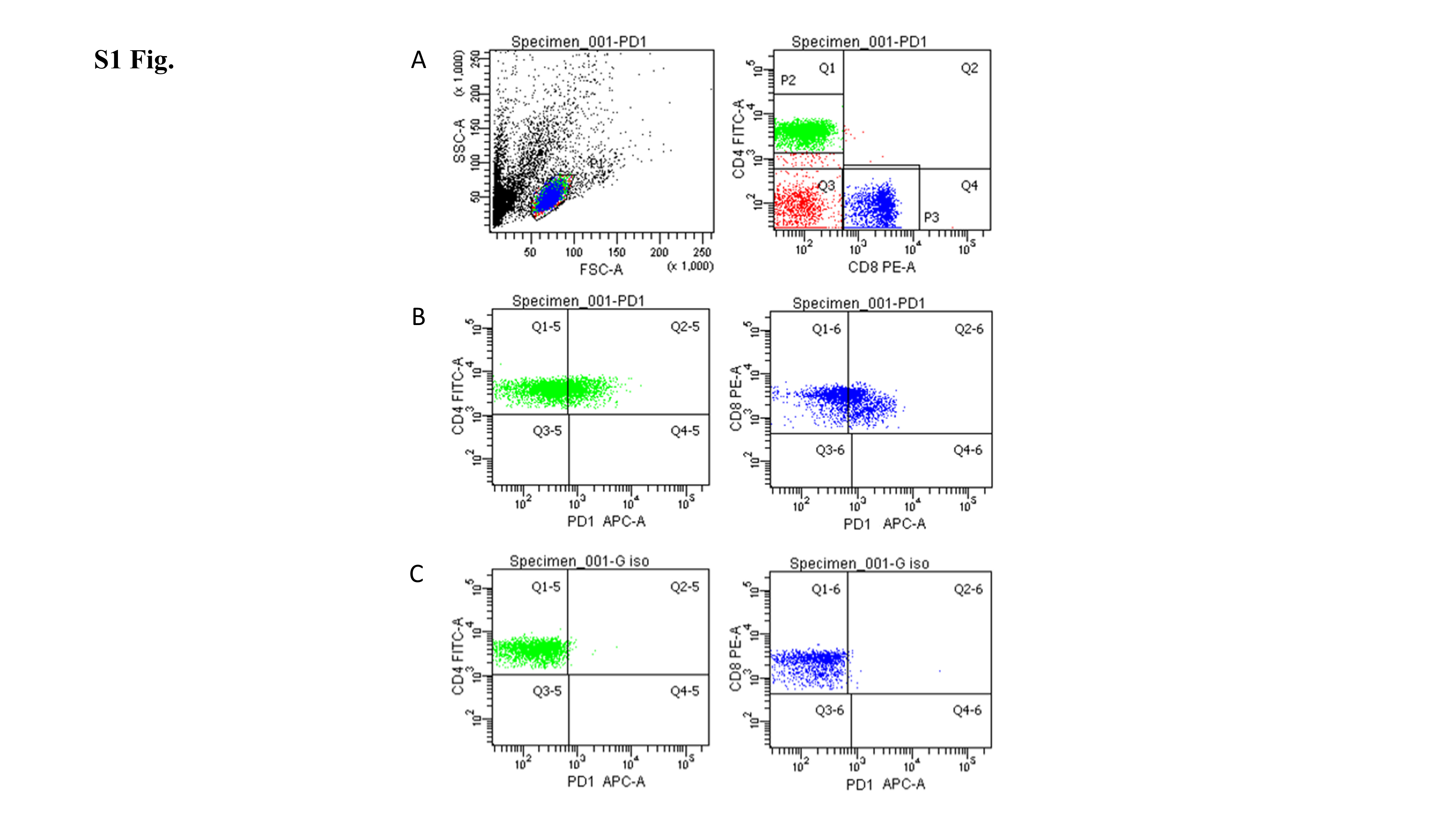

Supplement: S1 Fig — A representative sample of forward versus side scatter identified the predominant lymphocyte population captured in region P1. The proportions of CD4 (P2) and CD8 (P3) cells in P1 are indicated (A). The proportions of PD-1 expression cells in P2 and P3 are indicated in the middle panels (B). The under panels show each isotype control (C). SSC, side scatter; FSC, forward scatter; PE, phycoerythrin; FITC, fluorescein isothiocyanate; APC, allophycocyanin. (TIF) [file pone.0201222.s001.tif]

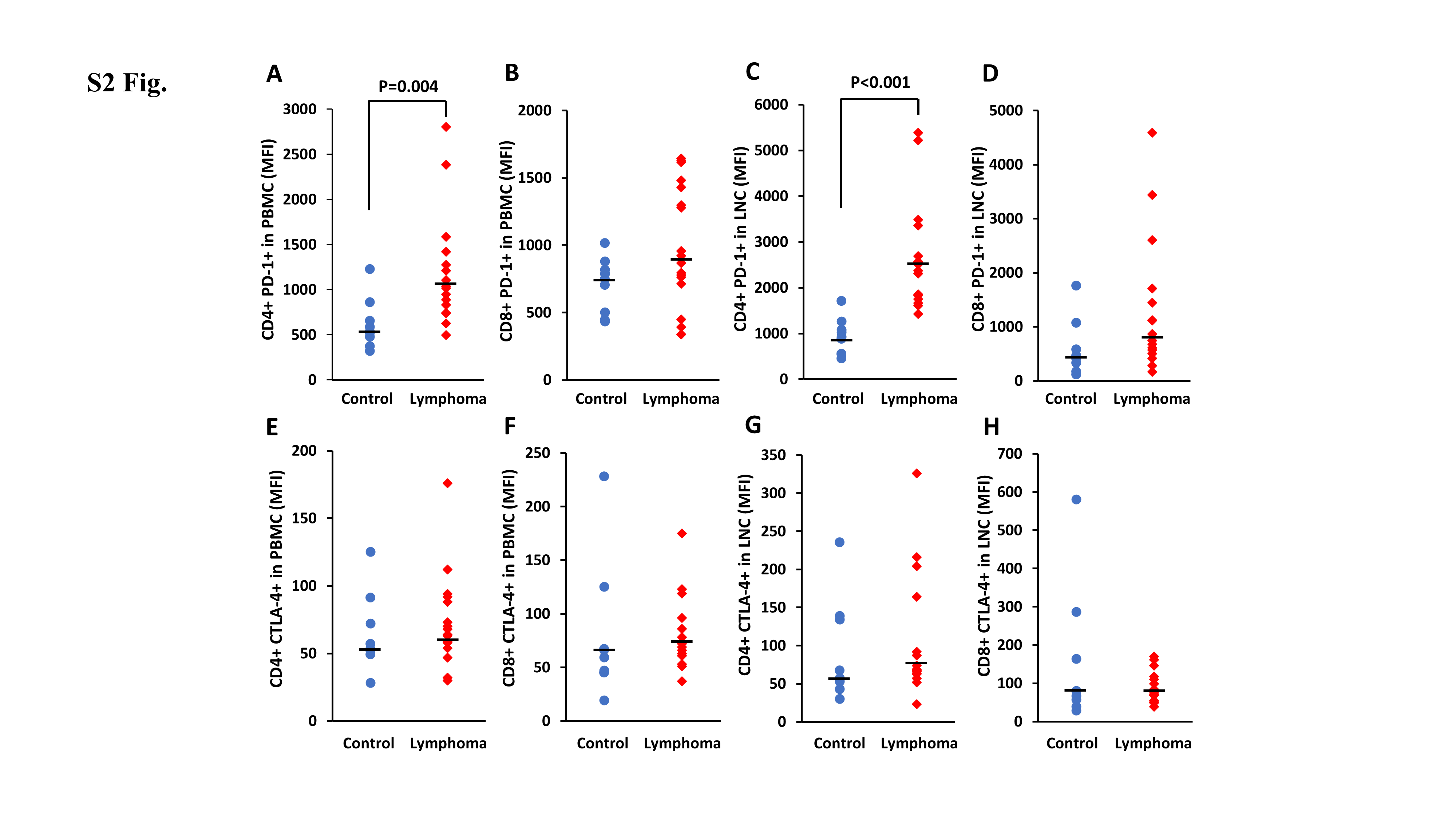

Supplement: S2 Fig — Each dot represents a patient and the bar represents the median. P values are shown. (TIF) [file pone.0201222.s002.tif]
